# Association Between DPP-4 Inhibitors and Events of Colorectal and Liver Cancers in Patients With Diabetes Receiving Second-Line Agents: A Nested Case-Control Study

**DOI:** 10.3389/fonc.2022.840142

**Published:** 2022-05-06

**Authors:** Chu-Lin Chou, Shu-Hui Juan, Ching-Hao Li, Hsi-Hsien Chen, Chih-Chin Kao, Li-Ying Chen, Li-Nien Chien, Te-Chao Fang

**Affiliations:** ^1^ Division of Nephrology, Department of Internal Medicine, Shuang Ho Hospital, Taipei Medical University, New Taipei City, Taiwan; ^2^ Division of Nephrology, Department of Internal Medicine, School of Medicine, College of Medicine, Taipei Medical University, Taipei, Taiwan; ^3^ Taipei Medical University Research Center of Urology and Kidney, Taipei Medical University, Taipei, Taiwan; ^4^ Department of Physiology, School of Medicine, College of Medicine, Taipei Medical University, Taipei, Taiwan; ^5^ Graduate Institute of Medical Sciences, College of Medicine, Taipei Medical University, Taipei, Taiwan; ^6^ Division of Nephrology, Department of Internal Medicine, Taipei Medical University Hospital, Taipei Medical University, Taipei, Taiwan; ^7^ Health Data Analytics and Statistics Center, Office of Data Science, Taipei Medical University, Taipei, Taiwan; ^8^ School of Health Care Administration, College of Management, Taipei Medical University, Taipei, Taiwan

**Keywords:** DPP-4 inhibitors, colorectal cancer, liver cancer, second-line agents, type 2 diabetes mellitus

## Abstract

**Objective:**

Plasma dipeptidyl peptidase-4 (DPP4) levels were significantly lower in patients with colorectal and liver cancers, and animal studies also showed DPP4 inhibitors (DPP4is) have procarcinogenic effects in colorectal cancer. Until now, whether DPP4is therapy affects the progression of liver cancer and colorectal cancer in patients with T2DM has not been well investigated. We investigated the association between cumulative defined daily dose (cDDD) of DPP4is exposure and risks of liver and colorectal cancers in patients with type 2 diabetes.

**Materials and Methods:**

We identified 268,520 patients with diabetes receiving DPP4is as second-line agents between March 1, 2009, and December 31, 2013, from Taiwan’s National Health Insurance Research Database, Taiwan Cancer Registry, and National Death Registry of Taiwan. The amount of DPP4is were divided into three groups (low, medium, and high) based on the interquartile range of the cDDD of the DPP4is.

**Results:**

The data showed that the low cDDD of DPP-4is was associated with a reducing risk of colorectal cancer [adjusted odds ratio (OR), 0.49; 95% CI, 0.32–0.75; P=0.001]. However, the high cDDD of DPP-4is was associated with an increasing risk of colorectal cancer (adjusted OR, 1.86; 95% CI, 1.32–2.61; P<0.001). No association between DPP4is use and liver cancer risk was observed.

**Conclusions:**

This nested case study revealed a J-shaped association between the cDDD of DPP-4is and colorectal cancer risk, but not liver cancer risk. Therefore, the effects of long-term DPP4is use on colorectal cancer risk warrant further study.

## Introduction

Type 2 diabetes mellitus (T2DM), which is associated with a high risk of organ dysfunction and high mortality, poses a high economic burden worldwide (1). In patients with T2DM managed through lifestyle modification, metformin monotherapy is recommended as the initial therapy (2). After metformin monotherapy fails to achieve target glucose control, a second-line agent may be administered, and the agent prescribed depends on the risk-benefit profile of individual agents.

Among oral antidiabetic drugs, dipeptidyl peptidase-4 inhibitors (DPP4is) are a new and promising class of second-line therapy (3). Recent research showed that the incretin-based insulinotropic therapeutic agent of the DPP4is provides favorable glucose control with negligible adverse effects, although hypoglycemia and pancreatitis were reported (4). Moreover, DPP4is are gaining popularity because of their glucose-lowering potential and possible pleiotropic benefits in patients with T2DM, such as lowering serum low-density lipoprotein (5), improving blood pressure (6, 7), and attenuating vascular remodeling processes (8).

Although DPP4is have pleiotropic benefits in addition to glucose-lowering effects, controversial results have been obtained regarding its tissue growth stimulation potential in animal studies (9, 10). Additionally, Javidroozi *et al. (* 11) reported that plasma dipeptidyl peptidase-4 (DPP4) levels were significantly lower in patients with cancers such as colorectal or liver cancer than in healthy subjects. Higher DPP4 levels were associated with higher survival in all cancers combined (11). Therefore, long-term DPP4is use has raised concerns regarding the risks of liver and colorectal cancer in patients with T2DM. Additionally, T2DM is an independent risk factor for liver cancer (12) and colorectal cancer (13). For example, an observational study including 388,619 person-years of follow-up between 1 January 2007 and 31 March 2015 from the UK Clinical Practice Research Datalink showed that DPP4is inhibitors tended to raise colorectal cancer incidence, although there was no significant difference in statistics (hazard ratio = 1.2, 95% CI = 1.0–1.5) (14). Furthermore, a recent meta-analysis of randomized clinical trials suggested it is mandatory to investigate further the associations between (1) DPP4is and colorectal cancer, and (2) DPP4is and liver cancer risk (15). Until now, whether DPP4is therapy affects the progression of liver cancer and colorectal cancer in patients with T2DM is inconclusive. Thus, in this nested case-control study, we investigated the association between DPP4is exposure and risks of liver cancer and colorectal cancer in patients with T2DM receiving second-line agents using data between March 1, 2009 and December 31, 2013 from the National Health Insurance Research Database (NHIRD), Taiwan Cancer Registry (TCR), and National Death Registry (NDR) of Taiwan.

## Materials and Methods

### Data Sources

This nested case-control study was conducted using data from the NHIRD, TCR, and NDR between March 1, 2009, and December 31, 2013. The NHIRD is a population-based claims database provided by the National Health Insurance Administration and managed by the Health and Welfare Data Science Center, Ministry of Health and Welfare, Executive Yuan, Taiwan. The NHI program covers more than 99% of residents of Taiwan. The NHIRD contains reliable clinical data that are utilized for population-based cohort studies in Taiwan (16, 17). It is one of the highest-quality databases worldwide (17) and is widely used for longitudinal cohort studies (18–28). The NHIRD contains data on drug prescriptions, diagnoses, and basic medical and demographic characteristics. In this study, diseases diagnoses were coded according to the International Classification of Disease, Ninth Revision, Clinical Modification (ICD-9-CM). Each patient has a unique encrypted identifier that can be linked to the TCR and NDR. Both the TCR and NDR are population-based registries, and detailed information on the completeness and accuracy of data from the NHIRD, TCR, and NDR has been provided and widely used for longitudinal cohort studies in previous studies (29, 30).

### Study Cohort

This study included patients with T2DM who were receiving metformin monotherapy as first-line treatment and initiating second-line therapy (add-on or switching) between March 1, 2009, and December 31, 2013. In Taiwan, the first DPP4is was launched on March 1, 2009, and was as second-line therapy for diabetes when metformin monotherapy fails to achieve glucose control or when metformin is contraindicated for specific patients, such as patients with chronic kidney disease. The exclusion criteria were as follows: 1) not a citizen of Taiwan or missing sex information, 2) diagnosed with cancer and human immunodeficiency virus before receiving a second-line agent, 3) diagnosed with cancer other than liver and colorectal cancer, and 4) without a minimum of 2 years of data available following initiation of second-line therapy.

### Patient and Control Selection

Patients diagnosed with liver cancer [International Classification of Diseases for Oncology, Third Edition (ICD-O-3) code C22] or colorectal cancer (ICD-O-3 codes C18, C19, C20, and C21) were identified from the TCR, and the date of cancer diagnosis was defined as the index date. Only patients with liver and colorectal cancers were included because T2DM is an independent risk factor for liver cancer (12) and colorectal cancer (13). Each patient was matched to four controls by sex, age (+/− 1 year), and follow-up period using an incidence density sampling approach, which involves matching a patient to control at risk of the disease at the time the patient is diagnosed with the disease. Because controls did not have cancer, they were assigned a date for a pseudo-cancer event, which corresponded to the index date of their matched patients (referred to as the index date hereafter). This approach enabled both patients and controls to be observed for similar periods, eliminating the bias caused by differences in the time frame.

### DPP4is Exposure

We selected a nested case-control design for this study. The primary reason for choosing a nested case-control design was because the DPP4 was a time-varying exposure; it may be likely that the patients who were DPP4 users in the first three months of the study became non-users during the other period of follow-up. Thus, we selected a nested case-control study design. Also, this study approach has several advantages compared to the standard case-control design: (1) cases and controls are sampled from the same population, (2) exposures are measured before the outcome occurs, and (3) cases can be matched to controls at the time (e.g., age) of the outcome event. Also, this study approach has several advantages compared to the standard case-control design: (1) cases and controls are sampled from the same population, (2) exposures are measured before the outcome occurs, and (3) cases can be matched to controls at the time (e.g., age) of the outcome event (31).

DPP4is use was determined using prescription claims data. DPP4is exposure was measured based on the cumulative defined daily dose (cDDD) within 2 years before the index date of patients and controls. Of these, patients who had no claims data on DPP4is use within the 2-year observational period were considered nonusers. Among the users, we used the interquartile range (IQR) to divide patients into three DPP4is exposure groups, low cDDD group (cDDD < Q_1_), medium cDDD group **(**Q_1_ **≤** cDDD **<** Q_3_), and high cDDD group (cDDD **≥** Q_3_). The reason for such patient classification was the U-shaped distribution of patients with DPP4is use, and this nonlinear distribution was likely more effectively presented using multiple groups.

### Covariates

Baseline demographic and clinical characteristics recorded before the index date were obtained. Socioeconomic status (SES) was measured based on monthly income calculated from the insurance premium provided in patients’ enrollment profile, and SES was divided into six categories. ICD-9-CM disease diagnostic codes for previous or coexisting diseases and Anatomical Therapeutic Chemical (ATC) codes for medication are listed in **Table S1**. The comorbidities for liver and colorectal cancer were cholangitis, cholelithiasis, cholecystitis, cirrhosis of liver, alcoholic liver disease, chronic nonalcoholic liver disease, hepatitis B, hepatitis C, inflammatory bowel disease, adenomatous polyposis, peptic ulcer, gastroesophageal reflux disease (GERD), cardiovascular disease, and hyperlipidemia. To quantify the severity of comorbidities, we used a modified Charlson–Deyo comorbidity index (CCI) without considering uncomplicated diabetes as a proxy measure. The prescribed medications included diabetic and nondiabetic medications. The major classes of diabetic medications were metformin, thiazolidinedione, sulfonylureas, alpha-glucosidase inhibitors, and insulin. The nondiabetic medications included angiotensin-converting enzyme inhibitors/angiotensin receptor blockers (ACEIs/ARBs), beta-selective blockers, diuretics, calcium channel blockers (CCBs), antiplatelet drugs, statins, nonsteroidal anti-inflammatory drugs (NSAIDs), and steroids.

### Statistical Analysis

We used standardized mean difference (SMD) to evaluate the balance of baseline characteristics between patients and controls. An imbalance was defined as an absolute value of >0.1. Stepwise conditional logistic regression was used to estimate the crude odds ratios (ORs), adjusted ORs, and 95% confidence intervals (CIs) for the association between DPP4is use and the risk of liver or colorectal cancer. We also performed subgroup analysis to examine further the association between DPP4is use and cancer in different age groups, male and female patients, and those receiving different diabetic agents. Statistical analyses were performed using SAS/STAT (version 9.3; SAS Institute, Cary, NC, USA) and STATA 12 (Stata Corp, College Station, TX, USA). A *P* value of <0.05 was considered statistically significant.

## Results

### Sample Size

**Figure 1:**
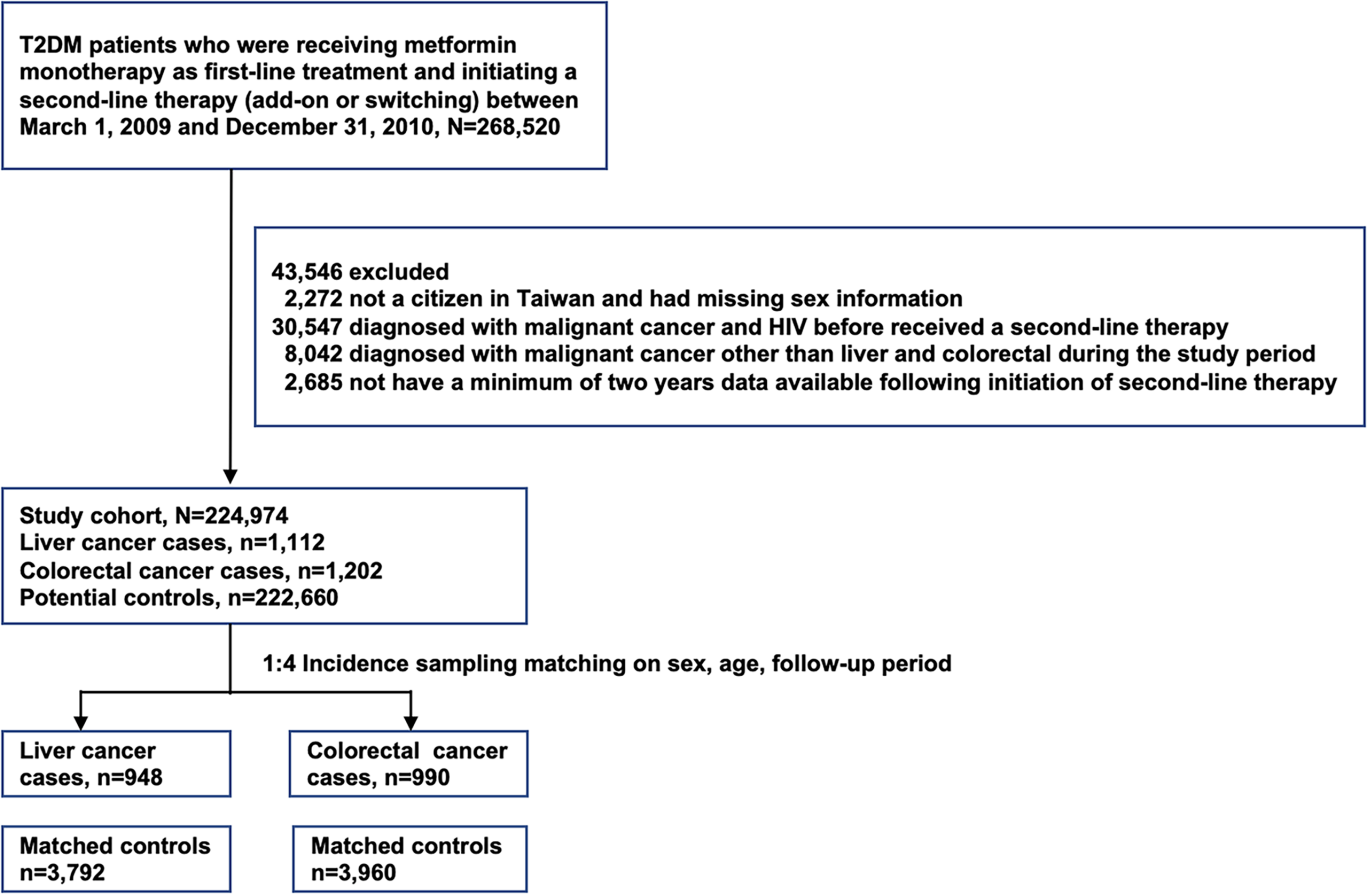
Flowchart of enrollment of patients with T2DM.

Initially, 224,974 patients with T2DM receiving second-line therapy were eligible for the study, and this nested case-control study comprised 948 and 990 patients with liver and colorectal cancer who were matched to 3,792 and 3,956 matched controls, respectively (**Figure 1**).

### Baseline Characteristics

**Table 1** presents the baseline characteristics of patients with liver (cohort 1) and colorectal cancer (cohort 2) and their matched controls. The mean age of the two cohorts was 63.4 years (standard deviation [SD]: 10.7 years). Patients with liver cancer were more likely to have higher CCI than matched controls, but this trend was not observed in patients with colorectal cancer. Patients with liver cancer were more likely than controls to have comorbidities related to bile and the liver, such as cholangitis, cholelithiasis, cirrhosis of liver, alcoholic liver disease, hepatitis B, and hepatitis C. Moreover, patients with liver cancer were more likely than controls to have adenomatous polyposis, peptic ulcer, GERD, and cardiovascular disease. Patients with colorectal cancer were more likely than controls to have peptic ulcer, GERD, hyperlipidemia, hypertension, and cardiovascular disease. Regarding previous or coexisting medication use, both groups of patients with cancer were more likely than controls to be prescribed with diuretics, NSAIDs, metformin, sulfonylureas, and insulin.

**Table 1:** Characteristics of patients with liver and colorectal cancer and age- and sex-matched controls among patients with T2DM receiving second-line therapy.

	Cohort 1					Cohort 2				
	Patients with liver cancer		Matched controls			Patients with colorectal cancer		Matched controls		
	*n*	(%)	*n*	(%)	SMD	*n*	(%)	*n*	(%)	SMD
**Sample size, *N***	948	(100.0)	3,792	(100.0)		990	(100.0)	3,960	100.0	
**Age (years), mean (SD)**	63.4	(10.7)	63.4	(10.7)		63.4	(10.7)	63.4	(10.7)	
**<45**	38	(4.0)	145	(3.8)	0.010	20	(2.0)	86	(2.2)	0.011
**≧45 and < 65**	467	(49.3)	1,890	(49.9)	0.012	409	(41.3)	1,631	(41.2)	0.003
**≧65**	443	(46.7)	1,757	(46.3)	0.008	561	(56.7)	2,243	(56.6)	0.001
**Male, yes**	675	(71.2)	2,700	(71.2)	0.008	637	(64.3)	2,545	(64.3)	0.001
**Socioeconomic status**										
**1 (highest)**	59	(6.2)	152	(4.0)	0.101	58	(5.9)	168	(4.2)	0.073
**2**	89	(9.4)	252	(6.7)	0.101	86	(8.7)	268	(6.8)	0.072
**3**	342	(36.1)	1,659	(43.8)	0.157	414	(41.8)	1,664	(42.1)	0.005
**4**	407	(42.9)	1,413	(37.3)	0.116	382	(38.6)	1,511	(38.2)	0.008
**5**	37	(3.9)	183	(4.8)	0.045	41	(4.1)	206	(5.2)	0.050
**6 (lowest)**	14	(1.5)	133	(3.5)	0.131	9	(0.9)	139	(3.5)	0.178
**Modified CCI**										
**0**	64	(6.8)	1,030	(27.2)	0.565	327	(33.0)	1,003	(25.3)	0.170
**1–2**	508	(53.6)	1,531	(40.4)	0.267	454	(45.9)	1,632	(41.3)	0.094
**≧3**	376	(39.7)	1,231	(32.4)	0.150	209	(21.1)	1,325	(33.4)	0.280
**Previous or coexisting diseases, yes**										
**Cholangitis**	18	(1.9)	22	(0.6)	0.119	6	(0.6)	45	(1.1)	0.057
**Cholelithiasis**	84	(8.9)	109	(2.9)	0.257	40	(4.0)	113	(2.9)	0.065
**Cholecystitis**	14	(1.5)	37	(1.0)	0.046	7	(0.7)	28	(0.7)	0.000
**Cirrhosis of liver**	511	(53.9)	450	(11.9)	1.000	33	(3.3)	298	(7.5)	0.186
**Alcoholic liver disease**	129	(13.6)	283	(7.5)	0.201	16	(1.6)	170	(4.3)	0.159
**Chronic nonalcoholic liver disease**	17	(1.8)	31	(0.8)	0.086	10	(1.0)	22	(0.6)	0.052
**Hepatitis B**	290	(30.6)	166	(4.4)	0.735	32	(3.2)	105	(2.6)	0.034
**Hepatitis C**	309	(32.6)	192	(5.0)	0.752	14	(1.4)	111	(2.8)	0.097
**Inflammatory bowel disease**	3	(0.3)	16	(0.4)	0.017	11	(1.1)	15	(0.4)	0.085
**Adenomatous polyposis**	38	(4.0)	14	(0.4)	0.251	6	(0.6)	7	(0.2)	0.069
**Peptic ulcer**	283	(29.9)	715	(18.9)	0.258	240	(24.2)	726	(18.3)	0.145
**GERD**	114	(12.0)	304	(8.0)	0.134	121	(12.2)	280	(7.1)	0.175
**Cardiovascular disease**	209	(22.0)	1,186	(31.3)	0.210	283	(28.6)	1,380	(34.9)	0.135
**Hyperlipidemia**	226	(23.8)	847	(22.3)	0.036	378	(38.2)	825	(20.8)	0.387
**Hypertension**	366	(38.6)	1,535	(40.5)	0.038	300	(30.3)	1,534	(38.7)	0.178
**Previous or coexisting medications, yes**										
**ACEI/ARB**	453	(47.8)	1,796	(47.3)	0.008	541	(54.6)	1,903	(48.1)	0.132
**Beta-2 blocker**	417	(44.0)	1,443	(38.1)	0.121	386	(39.0)	1,456	(36.8)	0.046
**Diuretic**	539	(56.9)	1,692	(44.6)	0.247	399	(40.3)	1,829	(46.2)	0.119
**CCB**	476	(50.2)	1,879	(49.6)	0.013	564	(57.0)	1,978	(50.0)	0.141
**Antiplatelet drug**	315	(33.2)	1,557	(41.0)	0.163	389	(39.3)	1,691	(42.7)	0.069
**Statin**	186	(19.6)	862	(22.8)	0.076	324	(32.7)	910	(23.0)	0.219
**NSAID**	729	(76.9)	2,370	(62.5)	0.317	721	(72.8)	2,340	(59.1)	0.293
**Steroid**	384	(40.5)	1,572	(41.5)	0.019	437	(44.1)	1,646	(41.6)	0.052
**Other OADs**										
**Metformin**	470	(49.6)	1,286	(33.9)	0.322	480	(48.5)	1,271	(32.1)	0.339
**Thiazolidinedione**	57	(6.0)	191	(5.0)	0.043	51	(5.2)	172	(4.3)	0.038
**Sulfonylureas**	443	(46.7)	1,279	(33.7)	0.267	427	(43.1)	1,248	(31.5)	0.242
**Alpha-glucosidase inhibitor**	118	(12.4)	384	(10.1)	0.073	106	(10.7)	353	(8.9)	0.060
**Insulin**	377	(39.8)	1,019	(26.9)	0.276	448	(45.3)	1,020	(25.8)	0.416

*SMD, standardized mean difference = difference in means or proportions divided by standard error, imbalance defined as absolute value greater than 0.1.

ACEI, angiotensin-converting enzyme inhibitor; ARBs, angiotensin receptor blockers; CCB, calcium channel blockers; CCI, Charlson–Deyo comorbidity index; GERD, gastroesophageal reflux disease; OADs, oral antidiabetic drugs; NSAID, nonsteroidal anti-inflammatory drug; SD, standard deviation; T2DM, type 2 diabetes mellitus.

### Association of DPP4is Use and Risks of Liver and Colorectal Cancer

**Table 2** presents the association between DPP4is use and the risk of liver cancer in patients with T2DM receiving second-line therapy. Patients were divided into DPP4is exposure groups according to the IQR of the cDDD. During the 2-year observation period, 17.9% of patients with liver cancer was DPP4is users and 16.0% of control patients were DPP4is users, respectively. When we compared patients with liver cancer with their matched controls, there was no association between DPP4is use and liver cancer risk.

**Table 2:** Association between DPP4is exposure and liver cancer risk among patients with T2DM receiving second-line therapy.

	Patients with liver cancer		Matched controls		Crude OR (95% CI)	*P*	Adjusted* OR (95% CI)	*P*
	*N*	(%)	*N*	(%)				
**Sample size**	948	(100.0)	3,792	(100.0)				
**DPP4is exposure group (cDDD within 2 years)**								
**Nonuser (cDDD=0)**	778	(82.1)	3,184	(84.0)	1.00 (Ref.)		1.00 (Ref.)	
**Low cDDD group (cDDD < 52)**	35	(3.7)	160	(4.2)	0.90 (0.62–1.32)	0.593	0.79 (0.48–1.29)	0.337
**Medium cDDD group (52 ≤ cDDD < 414)**	88	(9.3)	301	(8.0)	1.20 (0.93–1.54)	0.159	0.91 (0.64–1.28)	0.580
**High cDDD group (cDDD ≥ 414)**	47	(5.0)	147	(3.9)	1.32 (0.94–1.86)	0.113	1.13 (0.71–1.79)	0.602

*Adjustment for the covariates listed in **Table 1**.

DPP4is, dipeptidyl peptidase-4 inhibitors; CI, confidence interval; cDDD, cumulative defined daily dose; OR, odds ratio; Ref, reference group; T2DM, type 2 diabetes mellitus.

As shown in **Table 3**, the dose-response effect of DPP4is for colorectal cancer was J-shaped, indicating the dual effects of DPP4is. The low cDDD group of DPP4is exposure was associated with a reducing risk of colorectal cancer in patients with colorectal cancer than in their matched controls (adjusted OR = 0.49, 95% CI = 0.32–0.75, *P* = 0.001). By contrast, the high cDDD group of DPP4is exposure was associated with an increasing risk of colorectal cancer in patients with colorectal cancer than in their matched controls (adjusted OR = 1.86, 95% CI = 1.32–2.61, *P* < 0.001). No association between PP4is use and colorectal cancer risk was observed in the medium cDDD group.

**Table 3:** Association between DPP4is exposure and colorectal cancer risk among patients with T2DM receiving second-line therapy.

	Patients with colorectal cancer		Matched controls		Crude OR (95% CI)	*P*	Adjusted* OR (95% CI)	*P*
	*N*	(%)	*N*	(%)				
**Sample size**	990	(100.0)	3,960	(100.0)				
**DPP4is exposure group (cDDD within 2 years)**								
**Nonuser (cDDD=0)**	790	(79.8)	3,321	(83.9)	1.00 (Ref.)		1.00 (Ref.)	
**Low cDDD group (cDDD < 56)**	30	(3.0)	189	(4.8)	0.68 (0.46–1.01)	0.057	0.49 (0.32–0.75)	0.001
**Medium cDDD group (56 ≤ cDDD < 420)**	96	(9.7)	316	(8.0)	1.30 (1.02–1.66)	0.037	0.91 (0.69–1.20)	0.515
**High cDDD group (cDDD ≥ 420)**	74	(7.5)	134	(3.4)	2.34 (1.74–3.15)	<0.001	1.86 (1.32–2.61)	<0.001

*Adjustment for the covariates listed in **Table 1**.

The abbreviations as in **Table 2**.

### Subgroup Analysis of High cDDD Group for the Association Between DPP4is Use and Risk of Colorectal Cancer in Patients With T2DM Initiating Second-Line Therapy

**Figure 2:**
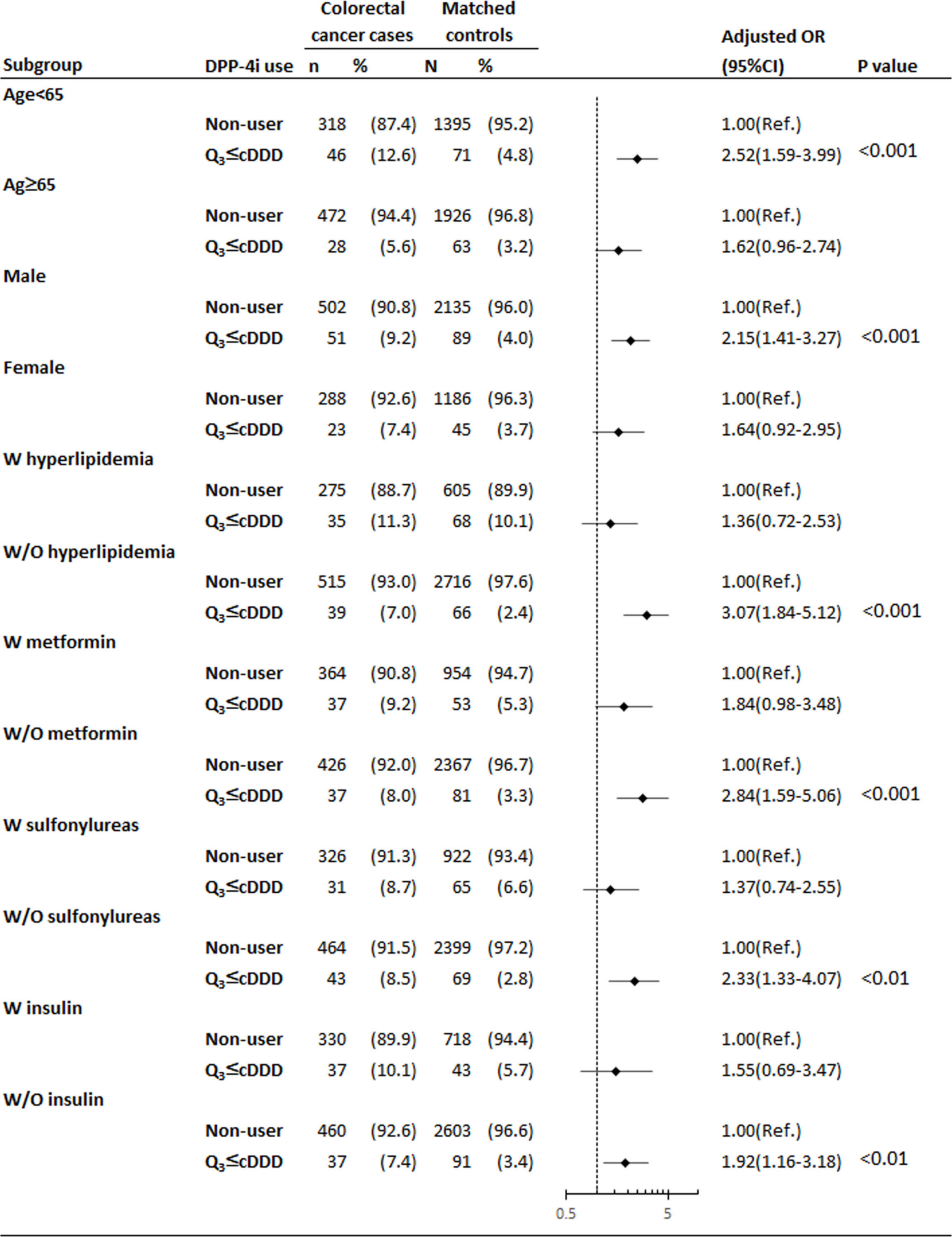
Subgroup analysis of high cDDD group for the association between DPP4is exposure and risk of colorectal cancer among patients with T2DM. *Adjustment for covariates listed in **Table 1**, including age, socioeconomic status, modified CCI, previous and coexisting medical conditions (cholangitis, cholelithiasis, cholecystitis, cirrhosis of liver, alcoholic liver disease, chronic nonalcoholic liver disease, hepatitis B, hepatitis C, inflammatory bowel disease, adenomatous polyposis, peptic ulcer, GERD, cardiovascular disease, hyperlipidemia, and hypertension), prescribed medications before the index date of cancer diagnosis (ACEIs/ARBs, beta-2 blockers, diuretics, CCBs, antiplatelet drugs, statin, NSAIDs, and steroids) and other OADs (metformin, thiazolidinedione, sulfonylureas, alpha-glucosidase inhibitor, and insulin). *ACEIs* angiotensin-converting enzyme inhibitors, *ARBs* angiotensin receptor blockers, *CCBs* calcium channel blockers, *CCI* Charlson–Deyo comorbidity index, *CI* confidence interval, *DPP4is* dipeptidyl peptidase-4 inhibitors, *NSAIDs* nonsteroidal anti-inflammatory drugs, *OADs* oral antidiabetic drugs, *OR* odds ratio, *Ref.* reference group.

During the 2-year observational period, patients with colorectal cancer aged less than 65 years (**Figure 2**) were more likely to be exposed to the high cDDD group than their matched controls (adjusted OR = 2.52, 95% CI = 1.59–3.99). Similar results were found for patients who were male, without hyperlipidemia, and did not take metformin, sulfonylureas, or insulin. For example, among insulin nonusers, the adjusted OR of patients with colorectal cancer to their matched controls was 1.92 (95% CI = 1.16–3.18).

## Discussion

This study was the first to examine the association between the cDDD of DPP4is exposure and risks of liver and colorectal cancers in patients with type 2 diabetes mellitus receiving second-line agents. After multivariate adjustment and subgroup analysis, the major findings of our study are as follows. (1) Based on the prospective data from the Taiwan NHIRD, DPP4is exposure was associated with colorectal cancer risk, in patients with T2DM, and the dose-response effect of DPP4is for colorectal cancer was J-shaped, indicating the dual effects of DPP4is. (2) No association between DPP4is use and liver cancer risk was observed. (3) Subgroup analyses showed that among the high cDDD group, DPP4is use remained a risk factor for colorectal cancer in patients who were less than 65 years old, male, without hyperlipidemia, and did not take metformin, sulfonylureas, and insulin.

Controversial results have been obtained regarding the association of DPP4is use with the risk of colorectal cancer. As reported previously, there is a protective effect of metformin use on the incidence of colorectal cancer (32). In this nested case-control study, our findings showed, the adjust OR 1.84 (95%CI, 0.98-3.38) in patients with receiving DPP4i and metformin; on the contrary, there were significant findings of the adjust OR 2.84 (95%CI, 1.59-5.06) in patients with receiving DPP4i and without receiving metformin, as shown in **Figure 2**, indicating the tumor promoting effect of DPP4is might be masked by metformin in patients who received low dosed DPP4is. Furthermore, our study provided a novel finding that the dose-response curve of DPP4is exposure for colorectal cancer is J-shaped, indicating the dual effects of DPP4is. The low cDDD group was associated with a reducing risk of colorectal cancer (adjusted OR = 0.49, 95% CI = 0.32–0.75, *P* = 0.001). By contrast, the high cDDD group was associated with an increasing risk of colorectal cancer (adjusted OR = 1.86, 95% CI = 1.32–2.61, *P* < 0.001). Emerging studies show that antiangiogenic agents exhibit J-shaped or U-shaped dose-response curves (33), indicating inhibitory effects at a low dosage and stimulatory effects at a higher dosage. DPP4is have potent antiangiogenic activities, which are mediated through the suppression of DPP4 and plasminogen activator inhibitor-1 (34).

Few *in vitro* and *in vivo* studies have evaluated the effects of DPP4is on colorectal cancer, and the association between DPP4is use and colorectal cancer remains unclear (35–37). Amritha *et al.* (35) reported that sitagliptin exerted more potent inhibitory effects on HT-29 colon cancer cell growth than vildagliptin. However, Wang *et al.* (36) found that saxagliptin and sitagliptin could increase cell migration and invasion in SW480 and HCT116 colon cancer cell lines through the activation of nuclear factor E2-related factor 2. Femia *et al.* (37) investigated the effect of 15-week sitagliptin treatment on colon carcinogenesis in 1,2-dimethylhydrazine-induced colon cancer rats and found fewer precancerous lesions (manifesting as mucin-depleted foci) in the colorectum in rats treated with sitagliptin than in controls. Therefore, additional *in vitro* and *in vivo* experiments will be necessary to evaluate the effect of DPP4is dose and duration on cancer cells.

The Saxagliptin Assessment of Vascular Outcomes Recorded in Patients with Diabetes Mellitus-Thrombolysis in Myocardial Infarction (SAVOR-TIMI) 53 trial in 2016 reported that saxagliptin exerted a protective effect against colon cancer (hazard ratio = 0.51, 95% CI = 0.27–0.92, *P* = 0.026) (38). However, the sample size of patients with colon cancer in this trial was small (number of patients = 47). A meta-analysis of randomized clinical trials in 2017 showed the insignificant effect of DPP4is on the risk of colon cancer (MH-RR = 0.96, 95% CI = 0.71–1.31, *P* = 0.808) (15). Furthermore, a meta-analysis report in 2020 showed the results of DPP4is on the risk of colon cancer resembled the results of 2017 meta-analysis (39). Furthermore, in 2018, in a large population from the UK Clinical Practice Research Datalink, the results of 388,619 person-years of follow-up between 1 January 2007 and 31 March 2015 indicated the use of DPP4is inhibitors tended to raise colorectal cancer incidence, although there was no significant difference in statistics (hazard ratio = 1.2, 95% CI = 1.0–1.5) (14), however, there was no available data of association between different type DPP4is and colorectal cancer. These findings indicate the ambiguous effect of DPP4is on the inhibition of and stimulation of cancers, and the conflicting results may be because of variable dosage and follow-up duration among these studies. Thus, additional cohort studies should be conducted to examine the J-shaped association (related to antiangiogenesis) between the cDDD of DPP4is use and the risk of colon cancer.

In the present study, no association of DPP4is use and liver cancer risk was observed between patients with liver cancer and their matched controls (adjusted OR = 1.13, 95% CI = 0.71–1.79, *P* = 0.602). In a meta-analysis, Zhao *et al.* showed no significant association between DPP4is use and the risk of liver cancer in patients with T2DM (MH-RR = 1.02, 95% CI = 0.54–1.91, *P* = 0.96) (15).

The strengths of our study are the large sample size, the use of a national database, the consideration of the cumulative dose effect of drug use, and the subgroup analysis. Moreover, in this population-based observational study, multiple potential risk factors from the Taiwan NHIRD were adjusted for the analysis. Despite its strengths and novelty, this study has some limitations that require clarification. First, drug exposure was measured based on prescription records, which might not reflect the actual use of drugs. Second, the NHIRD lacks information on patients’ risk behavior and clinical characteristics, which might affect the findings. Third, although all potential risk factors were adjusted for in this population-based observational study, unmeasured factors might have still biased our observations; therefore, the results should be interpreted with caution. Finally, this study was limited to the Taiwanese population, a short follow-up period, and no breakdown of the types of DPP4is.

## Conclusion

In summary, the novel finding of this nested case-control study showed a J-shaped association between the cDDD of DPP4is and the risk of colorectal cancer. This study provided the possible effects of long-term DPP4is use on colorectal cancer in patients with T2DM. Therefore, the effects of long-term DPP4is use on colorectal cancer risk warrant further study.

## Data Availability Statement

The raw data supporting the conclusions of this article will be made available by the authors, without undue reservation.

## Ethics Statement

The studies involving human participants were reviewed and approved by the Joint Institutional Review Board of Taipei Medical University (TMU-JIRB No. N201712032), and the informed consent requirement was waived for this study. Written informed consent for participation was not required for this study in accordance with the national legislation and the institutional requirements.

## Author Contributions

CL-C, LN-C, and TC-F participated in study design. CL-C, LN-C, and TC-F were responsible for the analysis of data. CL-C and LN-C were responsible for drafting the manuscript. TC-F put forward the concepts of the study. All the authors revised the manuscript. All authors contributed to the article and approved the submitted version.

## Funding

The study was financially sponsored by Taipei Medical University Hospital, Taipei Medical University, Taiwan (107TMU-TMUH-03 and 110TMU-TMUH-17). The funder had no role in study design, data collection and analysis, publication decision, and manuscript preparation.

## Conflict of Interest

The authors declare that the research was conducted in the absence of any commercial or financial relationships that could be construed as a potential conflict of interest.

## Publisher’s Note

All claims expressed in this article are solely those of the authors and do not necessarily represent those of their affiliated organizations, or those of the publisher, the editors and the reviewers. Any product that may be evaluated in this article, or claim that may be made by its manufacturer, is not guaranteed or endorsed by the publisher.

## Supplementary Material

Supplementary Table 1 Disease diagnosis codes according to the international classification of diseases, ninth revision, clinical modification (ICD-9-CM) and prescribed medications classified based on anatomical therapeutic chemical (ATC) classification *ACEI* angiotensin-converting enzyme inhibitor, *ARB* angiotensin receptor blocker, *CCB* calcium channel blockers, *DDP4is* dipeptidyl peptidase-4 inhibitors, *GERD* gastroesophageal reflux disease, *NSAID* nonsteroidal anti-inflammatory drug.
