# Supplementary material for: Association Between DPP-4 Inhibitors and Events of Colorectal and Liver Cancers in Patients With Diabetes Receiving Second-Line Agents: A Nested Case-Control Study
Source: Front Oncol. 2022 May 6;12:840142. doi: 10.3389/fonc.2022.840142 (PMC9120816; doi:10.3389/fonc.2022.840142)
Supplement: Supplementary Table 1 — Disease diagnosis codes according to the international classification of diseases, ninth revision, clinical modification (ICD-9-CM) and prescribed medications classified based on anatomical therapeutic chemical (ATC) classification ACEI angiotensin-converting enzyme inhibitor, ARB angiotensin receptor blocker, CCB calcium channel blockers, DDP4is dipeptidyl peptidase-4 inhibitors, GERD gastroesophageal reflux disease, NSAID nonsteroidal anti-inflammatory drug. [file Table_1.docx]

Table S1 Disease diagnosis codes according to the international classification of diseases, ninth revision, clinical modification (ICD-9-CM) and prescribed medications classified based on anatomical therapeutic chemical (ATC) classification

| **Comorbidity/Medication** | **ICD-9-CM/ATC codes** |
| --- | --- |
| Cholangitis | 5761 |
| Cholelithiasis | 574 |
| Cholecystitis | 5750,5751 |
| Cirrhosis of liver | 5715,5716,5722-5724,5728,5730 |
| Alcoholic liver disease | 291,3030,3039,3050,5710-5713 |
| Chronic nonalcoholic liver disease | 5718 |
| Hepatitis B | 0702,0703,V0261 |
| Hepatitis C | 0707,07041,07044,07051,07054,V0262 |
| Inflammatory bowel disease | 555,556 |
| Adenomatous polyposis | 2112,2115-2117 |
| Peptic ulcer | 531-533 |
| GERD | 53011,53081 |
| Cardiovascular disease | 4273,410-414,39891,422,425,428,40201,40211, 40291,40401,40411,40491,40403,40413,40493, V421,440-444,447,557 |
| Hyperlipidemia | 272 |
| Hypertension | 401-405 |
| ACEI/ARB | C09 |
| Beta-2 blocker | C07 |
| Diuretic | C03 |
| CCB | C08 |
| Antiplatelet drug | B01AC |
| Statin | C10AA |
| NSAID | M01AA,M01AB,M01AC,M01AE,M01AG,M01AH, M01AX |
| Steroid | H02A,H02B,H02C |
| DPP4is | A10BH,A10BD07,A10BD08,A10BD10,A10BD11 |
| Metformin | A10BA |
| Thiazolidinedione | A10BG |
| Sulfonylureas | A10BB |
| Alpha-glucosidase inhibitor | A10BF |
| Insulin | A10A |

*ACEI* angiotensin-converting enzyme inhibitor, *ARB* angiotensin receptor blocker, *CCB* calcium channel blockers, *DDP4is* dipeptidyl peptidase-4 inhibitors, *GERD* gastroesophageal reflux disease, *NSAID* nonsteroidal anti-inflammatory drug
